# A Case of Puerperal Convulsions

**Published:** 1849

**Authors:** Bradley Noyes

**Affiliations:** Beaver Dam, Wisconsin


### ARTICLE IV.

*A Case of Puerperal Convulsions.* By BRADLEY NOYES M.D. of Beaver Dam, Wisconsin.

Mrs L., aged twenty eight years, a large, plethoric, robust woman, pregnant with her first child, was suddenly attacked on the 19th of March, 1849, at 2 o'clock A. M., with puerperal convulsions. There were no premonitory symptoms, except slight nausea and vomiting. I arrived there about four hours after the attack, and was informed by her attendants that she had had two fits. At that time she was extremely sick at the stomach, and vomited large quantities of glairy billious matter. She informed me that she thought herself only in the eighth month of her pregnancy; and there had been no labour pains. On examination found the os uteri had not commenced dilating. I gave her a small dose of solution of morphine, repeating it often until the irritability of the stomach and vomiting were checked. She was quiet and composed, except slight nausea and vomiting occasionally, for about six hours, when she was suddenly siezed with a violent convulsive fit. I immediately began to make preparations to bleed her. but in consequence of her peculiar local situation, (she was living in a very small, dark, board shantee,) and for the want of a suitable assistant, she had two more fits in almost immediate succession, before the operation could possebly be performed. I abstracted not less than a quart of blood from a large orifice in the arm, and gave her two common sized pills composed of pill. hyd. c. magnes. calc. On examination again per vaginum, found the os uteri had commenced dilating, and was satisfied that parturition had commenced. She was at this time, (2 o'clock P. M.) and had been since the third fit, nearly insensible, with stertorous breathing, except occasionally being restless and uneasy, which I have no doubt was owing to the contractions of the uterus, as is usually the case in the first stage of parturition.

The bleeding seemed to check the recurrence of the fits for a while, but within an hour they recurred again, and continued as often as every half hour, alternating with uterine contractions, and a most wild and furious look of countenance, and a violent tossing and throwing herself about, till about midnight when the head of the foetus had passed down in the pelvis. Her fits had become very severe and alarming, the convulsions were strong and very frequently repeated, her tongue lacerated by the teeth, blood flowing from the mouth, and she writhed, and turned and twisted herself in every possible manner. I became convinced that the only way to save her life was to remove the child dead or alive. An attempt was made to apply the forceps, but she turned and tossed herself about so, that at the first attempt, it was impossible to introduce them. At length, by the assistance of three persons, I succeeded in applying them, and soon delivered her of a living, healthy, female child about middle size. The placenta was readily removed. The insensibility still continued, with occasional spasms, when in about three fourths of an hour, the fits recurred again, and continued at short intervals for four or five hours, when on the repetition of the cathartic with two or three doses of ol. ricini, enemas and solution of morphine, they gradually subsided, and, after lying comatose a few hours, she suddenly started up in bed and attempted to step off on the floor, but she was arrested by her husband. Said she wanted to get the comb to comb her hair, which was handed her, and she immediately commenced combing. She was asked whether she knew any thing that had been going on, or when her child had been taken away from her, and she answered decidedly in the negative. On the second day the mother and child were getting along well.

